# Energy (calorie) labelling for healthier selection and consumption of food or alcohol

**DOI:** 10.1002/14651858.CD014845

**Published:** 2018-02-27

**Authors:** Natasha Clarke, Theresa M Marteau, Mark Pilling, Nia W Roberts, Susan A Jebb, Gareth J Hollands

## Background

### Description of the condition

Poor diets - high in saturated fats, free sugars and salt, and low in dietary fibre, fruits and vegetables - and the consumption of alcohol contribute to the global prevalence and burden of obesity and non-communicable diseases, including cardiovascular disease, diabetes and many cancers (NCD Countdown 2020; Rehm 2018; Sheron 2016). Worldwide, 2.8 million people are estimated to die annually as a result of obesity (EASO 2021; World Health Organization 2020a), with higher prevalence in the most deprived groups (Bann 2017; Health Survey for England 2018; NHS Digital 2019). There are also considerable economic costs associated with obesity (Tremmel 2017). For example, in the United Kingdom (UK), attributable National Health Service (NHS) costs are expected to reach an estimated 9.7 billion pounds sterling (GBP) by 2050 (Public Health England 2017). However, changing diet- and alcohol-related behaviours to halt and reverse rises in these potentially preventable diseases is difficult. While many people want to engage in behaviours that promote good health, most find it difficult to implement and maintain them (Sheeran 2016). This is in part due to physical environments that can exert considerable influence on our health-related behaviours, often with relatively little conscious engagement (Hollands 2016; Marteau 2012). Altering these environments may provide a catalyst for behaviour change (Das 2012; Marteau 2012). We have previously described a set of interventions to change health-related behaviours that involve altering the small-scale physical environment in which such behaviours are performed (Hollands 2013; Hollands 2017). This includes interventions that use words, numbers and symbols to convey specific information about food and alcohol products and their selection and consumption. Energy (or calorie) labelling and broader nutritional labelling are two key examples.

### Description of the intervention

Nutritional labels provide information about the nutritional content of a food or drink (e.g. the energy content or amount of fat, sugars or salt). The type of information provided varies across countries, for example, with regard to which nutrients are presented and the format in which this information is communicated. The current review focuses specifically on one type of these labels, energy (or calorie) labelling, which is the most frequent focus of both research and policy implementation (Department of Health and Social Care 2020b; Robinson 2019; Zlatevska 2018). We consider energy (or calorie) labelling as it applies to both food products (including non-alcoholic drinks) and alcohol products. We define energy labelling as a label that clearly quantifies the energy or calorie value of a product; that is, in kilocalories or other equivalent metric (e.g. kilojoules).

### Energy labelling of food

Pre-prepared, often pre-packaged, foods form a substantial part of dietary intake in many parts of the world. These food products are often complex items, with a mix of ingredients that make it difficult for consumers to know their nutritional or energy content. Many countries have implemented mandatory nutritional labelling, including the United Kingdom, United States of America, Australia, New Zealand, Canada, Mexico, Argentina, Brazil, Chile, Colombia, Ecuador, Paraguay, Uruguay, Israel, Gulf Cooperation Council members, Japan, India, China, Hong Kong, South Korea, Malaysia, Taiwan and Thailand (European Food Information Council 2018). In the European Union, Food Information Regulations made ingredient and nutrition declarations mandatory for most pre-packaged foods from December 2016 (European Union 2011). These regulations stipulate that manufacturers must provide nutritional information in a consistent format for most pre-packaged foods, including information on energy content as well as on fat, saturated fat, carbohydrate, total sugars, protein and salt (expressed per 100 g or per 100 mL of the food product). Additionally, manufacturers are able to repeat information in ‘the principal field of vision’; in other words, on the front of the pack (Food and Drug Administration 2016). This is purely voluntary but, where provided, only information on energy or energy plus fat, saturated fats, sugars and salt can be given, either per 100 g or 100 mL, or per portion, or both. These front-of-pack nutritional labelling schemes have usually been designed to guide consumer choice and sometimes include an interpretative component, such as reference to daily intake guidelines or colour coding to indicate relative healthiness. These can supplement, but not replace, the mandatory, back-of-pack nutrition declarations. In the UK, for example, a voluntary front-of-pack scheme using red, amber and green colour coding according to nutrient content, is widely used (European Food Information Council 2018).

### Energy labelling of alcohol

Alcohol is energy dense (7.1 kcal/g) and the consumption of alcoholic drinks accounts for almost a tenth (8.4%) of the total energy intake of adult drinkers aged 19 to 64 in the UK (Public Health England 2016), estimated to be about 60% of the population (Statistica 2019). Most countries do not require the energy content to be displayed on alcohol products (World Health Organization 2018). In the EU, all alcoholic drinks above 1.2% alcohol by volume are exempt from mandatory nutrition labelling, but energy declarations can be made on a voluntary basis. Most products do not display this information. Drinkers’ knowledge of the energy content of alcoholic drinks is limited – with many people underestimating their energy content (Royal Society for Public Health 2014). Health advocacy organisations have called for the inclusion of calorie labelling on alcohol (Royal Society for Public Health 2018; World Health Organization 2017), and England’s most recent obesity strategy includes plans to consult on whether to make companies provide this information (Department of Health and Social Care 2020a).

### Menu energy labelling

In addition to labelling packaged foods and drinks, some countries have introduced labelling on menus. Mandatory energy labelling in restaurants was first introduced in the state of New York (USA) in 2008 (Dumanovsky 2011). In 2016, the US Food and Drug Administration (FDA) ’final rule’ for all states became effective, requiring that energy information be listed on menus and menu boards in chain restaurants with 20 or more locations, as well as in all vending machines (Food and Drug Administration 2016). Similarly, the Healthy Menu Choices Act 2015 came into force in 2017 in Ontario, Canada. Since 2011, some states of Australia have also implemented a labelling policy, requiring mandatory energy labelling on menus in fast food chains and on vending machines (Obesity Policy Coalition 2018). In England, a recently published consultation on mandating calorie information out-of-home means that, by 2021, businesses will be required to display energy labels alongside a declaration referring to recommended daily intake (Department of Health and Social Care 2020b).

**Figure 1:**
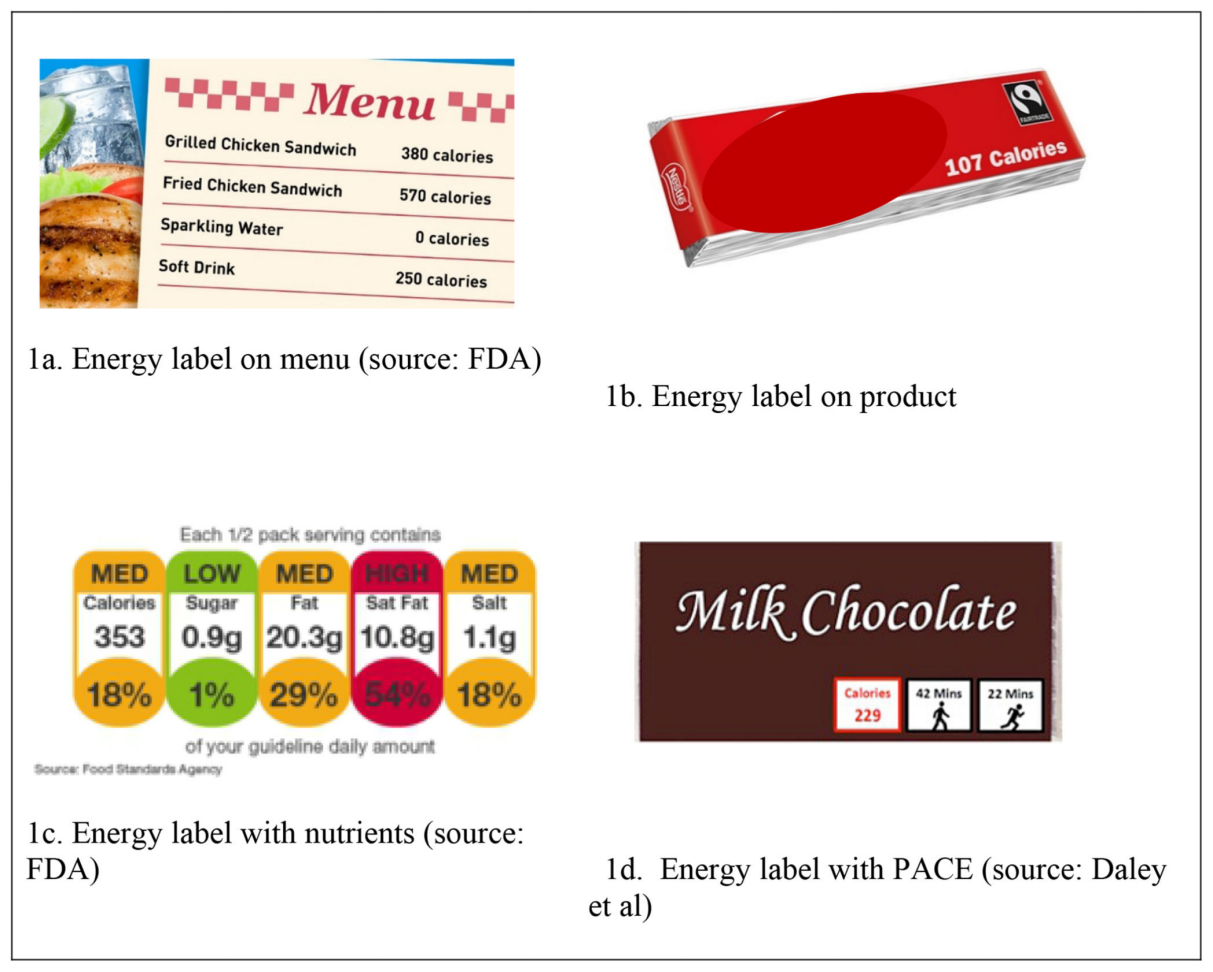
Examples of energy/calorie labels used in practice

In the absence of international agreements, there has been, and continues to be, considerable variation in both the information provided and the presentation format for energy and nutritional labelling. In terms of possible formats for energy labels specifically, labels may include numerical information on energy content, energy as a proportion of daily guidelines, colours (e.g. traffic light labelling) to indicate a product’s content relative to national guidance, or they may communicate energy in an alternative format, such as physical activity equivalents (see Figure 1 for example labels).

### How the intervention might work

**Figure 2:**
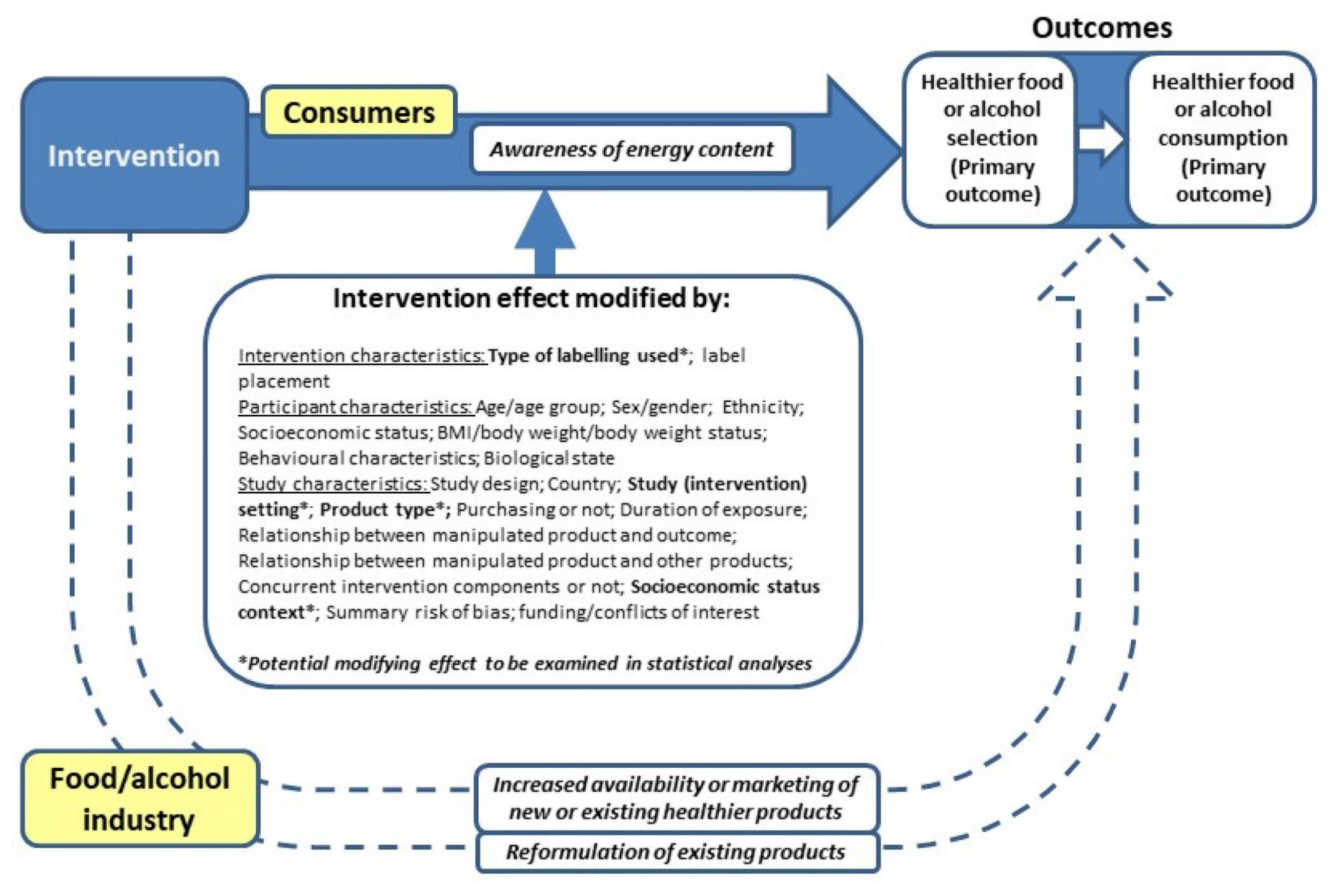
Logic model of processes by which nutritional labelling may have an impact on key behaviours. Blue shaded sections are the focus of the current review.

Energy labelling may impact on population health by encouraging the selection of healthier food and alcohol, with selection being a proximal determinant of healthier consumption (Cowburn 2005; Dobbs 2014; World Health Organization 2004; World Health Organization 2020a). Figure 2 presents a logic model of processes by which energy labelling may impact upon food and alcohol selection and consumption, which are determinants of health outcomes. Concerning direct effects on consumers, a central underlying mechanism may be via increased understanding of the energy content and related perceptions of healthiness of food and drink products. Possible key modifiers of any intervention effect include the setting in which people purchase the targeted products (e.g. a grocery store, fast food or other restaurant (Bollinger 2011; Harnack 2008)), the type of label used (e.g. Daley 2020), and the type of product that is labelled (e.g. Hollands 2015). Another potential modifier is socioeconomic status (SES), with obesity in high-income countries patterned by an individuals’ material and social resources (socioeconomic status (SES) or socioeconomic position (SEP)), reflected in levels of education, occupational status, family income, and area of residence (NHS Digital 2019; Winkleby 1992).

This review will include three planned subgroup analyses to investigate type of label, setting and SES as potential modifiers *(see* Subgroup analysis and investigation of heterogeneity). We have prioritised these because, first, it is widely acknowledged that labels differ in their presentation, and that this may lead to differential effects (e.g. see Daley 2020), and have implications for developing and implementing labelling interventions. Second, previous reviews have also shown differential effects of labelling interventions by study setting (e.g. Clarke 2020a), and this could have important implications for the implementation of labels in different real-world contexts. Finally, SES could modify any potential impact of energy labelling, and interventions that involve the provision of numeric or text-based information may be less effective in more deprived populations (Hollands 2016; Sarink 2016). An intervention should not widen further existing inequalities by having a greater effect in those of high SES, particularly given obesity rates are already patterned by SES. More consistent reporting of differential intervention effectiveness will help to build the evidence-base to test for so-called “Intervention-Generated-Inequalities” (IGI) (Lorenc 2013). Participants in a study are sometimes categorised in terms of material and social deprivation based on individual level characteristics or characteristics of where they reside (NHS Digital 2019; Winkleby 1992). Previous evaluations of differential effects of labelling by SES conclude that the current evidence is limited in both quantity and quality, and that further evaluation of differential effectiveness of labelling by SES is required (Sarink 2016). In accordance with methodological guidance on the importance of considering inequalities, incorporating study-level data on SES of participants is intended to enable us to interpret any differential effects through a health equity lens (Welch 2012).

This review focuses specifically on direct effects of energy labelling on people who select or consume the targeted food and alcohol products. However, as illustrated in Figure 2, there may also be indirect effects of implementing such interventions on people because industry may modify its actions or practices. For example, these modifications may include industry removing high energy items from sale, increasing the availability of lower energy options by encouraging the reformulation of products to reduce energy content, enabling a healthier nutritional profile on a visible label (Robinson 2021; Shangguan 2019), or changing the focus of marketing.

### Why it is important to do this review

The large and increasing burden of diet- and alcohol-related disease worldwide requires population level interventions to promote changes in behaviour. Although energy labelling has been implemented unsystematically in North America, Europe and Australasia, there remains no consensus on whether it is effective for achieving healthier selection and consumption of food and alcohol. There is a continued need for robust evidence to support decisions regarding the implementation of energy labelling and the development of food and alcohol policy and programmes globally. For example, the World Cancer Research Fund’s (WCRF) global ‘Nourishing’ framework includes a range of policy options to promote healthier eating and prevent obesity, including nutrition label standards and informing people about food and nutrition (World Cancer Research Fund 2021). A range of UK government reports (e.g. ‘Calorie reduction: The scope and ambition for action’ ((Public Health England 2018 (2018); ‘Childhood obesity: a plan for action, chapter 2’ (Department of Health and Social Care 2018 2018)) cited a previous version of this review (Crockett 2018). The meta-analytic effect size that Crockett and colleagues estimated in the review was used in calculations for the Department of Health’s impact assessment for its consultation on mandating out-of-home calorie labelling (Department of Health and Social Care 2020b). Furthermore, England’s recent obesity strategy outlines plans to introduce legislation to require large businesses, including restaurants, cafes and takeaways, to provide calorie labels (Department of Health and Social Care 2020a). Given the considerable research and policy interest in this area, updating this review is a priority.

The current evidence base to inform the type of label or format is uncertain. For example, the previous version of this Cochrane eview identified only a small body of studies with, at best, low certainty evidence for all review outcomes (Crockett 2018). Notably, it included only three randomised controlled trials (RCTs) examining purchasing in real-world field settings. We are aware of a number of new studies published since the original review, including multiple RCTs in real-world settings (Vasiljevic 2018; Vasiljevic 2019), and an increase in research into a relatively new form of energy label - PACE (Physical Activity Calorie Equivalent) labels (Daley 2020). Generating the most up-to-date and reliable estimate is therefore important, particularly considering effect size estimates from Crockett 2018 are being used in impact assessments for national-level policy.

The previous systematic review focused on nutritional labelling on food. For this updated review, we are narrowing our focus to energy labelling. The large majority of studies in the previous review included energy labelling as the sole component or as part of the broader nutritional labelling scheme. As previously mentioned, this form of labelling remains the principal focus of continued policy and research interest (Department of Health and Social Care 2020a). Concurrently, we are broadening the range of target products to include alcoholic drinks, reflecting recent policy interest in energy labelling on alcohol products (Royal Society for Public Health 2018). For example, England’s recent obesity strategy includes intentions to ensure companies provide calorie information on alcohol (Department of Health and Social Care 2020a).

## Methods

### Criteria for considering studies for this review

#### Types of studies

We will include randomised controlled trials (RCTs) or quasi-randomised controlled trials (Q-RCTs) with between-subjects (parallel group) or within-subjects (cross-over) designs that compare an energy labelling intervention with a no-label (or a label that is not health-, nutrition-, or energy content-related) control. We will include quasi-randomised studies, in which the randomisation sequence was not truly random (Reeves 2020), because of the difficulty of implementing true randomisation at an aggregate, population level. We will also include cluster-randomised controlled trials, when randomisation is by site (e.g. grocery store), providing the study includes at least two intervention sites and two control sites.

We will also include interrupted time series studies (ITSs) that compare selection or consumption before and after the implementation of calorie labelling. In line with the Cochrane Effective Practice and Organisation of Care (EPOC) group recommendations, we will only include ITS studies if they have a clearly defined time point at which the intervention occurred and at least three observation periods both before and after the intervention (EPOC 2017), wherein the authors have presented these data within a graph or analysed them using regression analysis, preferably using segmented regression, or both. Based on Cochrane recommendations, we will exclude studies that report only a simple pre- and post-intervention comparison (Cochrane Public Health Review Group 2011; EPOC 2017). We will also include controlled before-and-after (CBA) studies that measure selection or consumption before and after implementation of an intervention in non-randomised intervention and control groups. Such studies need to have at least two intervention sites and two control sites, and the characteristics of the different groups have to be similar. If we identify CBA designs that are eligible, members of the review team not involved in extracting data (SAJ, TMM, MP) will, blinded to study results, agree and specify criteria for comparability dependent on the specific context of that study. At outset, we would expect to require intervention and control sites to be well matched for at least type of site, geographic location, and population size and their demographic characteristics.

#### Types of participants

Adults or children selecting (with or without purchasing) or consuming food or drink will be eligible for inclusion. Selections will include those by an individual for their personal consumption, or for consumption by a small group that the individual belongs to, such as their family. Food or drink selections will include those from any retail outlet including grocery stores and other food stores, vending machines, cafeterias, bars, pubs, and both fast food and non-fast food restaurants.

#### Types of interventions

Eligible interventions will include energy labelling of a food (including non-alcoholic drinks) or an alcoholic drink product. Eligible labels must possess the two characteristics below – concerning necessary information and visibility - or they will be classed as incomplete and considered ineligible.

##### Information about energy content

The label provides information about energy contained in the product. Information is given specifying the absolute amount of energy contained in the product or in a serving size. The energy label is required to display numeric information about content; for example, calories in a meal/pack/serving/drink. Figure 1 shows example images of energy labelling, on products and on menus. We will exclude studies if only a relative or categorical descriptor is used; for example, lower/higher energy, low/high or an indication that it meets a certain threshold rather than exact amount. We will not consider warning labels about the health implications of a product’s energy content to be eligible labels (e.g. Clarke 2020a; Clarke 2020b). We will also exclude logos or general health claims providing a summary assessment of the healthiness or content of a product. We will exclude studies that purposefully mislabel the energy content of products to mislead or misinform participants, meaning that products in included studies must have their content accurately described.

##### Visibility

The energy labels need to be visible at the point of selection or consumption. In some cases, the label might be placed on the front of product packages or containers. In other cases, the energy label might not appear on, but rather alongside, the product. Examples include labels on a shelf where the products are being displayed in a grocery store, on the exterior of a vending machine selling snacks, on the counter from which the food is being served in a cafeteria, or on a restaurant menu from which food or drinks are being selected. We will exclude studies where information was provided separately, for example, on a company website.

As noted above, the intervention labelling group has to be compared with a no-labelling control group (or a label that is not health-, nutrition-, or energy content-related). Thus, we will exclude studies that only compare two or more different types of energy labelling schemes without a control group.

We will only include interventions that combine an energy label with other substantive discrete intervention components – either other types of (non-energy-related) labelling, or other interventions unrelated to labelling - if we can isolate the effect of an energy label. We will exclude studies that assess multiple intervention components that include energy labels but do not allow the effect of the latter to be isolated. For example, if an intervention group combined an energy label intervention and a pricing intervention, and this was compared to a control condition that did not include a pricing intervention, the specific effect of the energy label could not be estimated.

Intervention details will be ascertained through study reports – either text descriptions or images of the energy label, or both. We will contact study authors if there are insufficient details on the intervention or comparator groups to assess the study’s eligibility.

#### Types of outcome measures

Eligible studies have to assess an objectively measured behavioural outcome of food or drinks selected (with or without purchasing) or consumed (see more specific parameters below).

#### Primary outcomes

We will include four primary outcomes:

- food or drink selected with or without purchasing;
- alcoholic beverages selected with or without purchasing;
- food or drink consumed; and
- alcoholic beverages consumed.

These are outlined in detail below

##### Selection

We will consider healthier food and non-alcoholic drink selection to comprise a reduction in energy selected. Depending on how this is assessed, it could reflect either a total reduction in energy selected, or fewer higher energy products being selected. Where total energy selected is available, we will prioritise this over fewer higher energy products being selected.

We will consider healthier alcoholic drink selection to comprise a reduction in energy selected (total reduction in energy selected, or fewer higher energy products selected), or a reduction in the volume or units of alcohol selected, or both. For example, selection of lower strength alcoholic beverages would lead to fewer units of alcohol selected, while selection of fewer alcoholic beverages would lead to less energy, as well as volume of alcohol selected.

Studies are required to assess selection with or without purchasing either at the individual or population group level. Studies from real-world settings or laboratory settings will be included. In the context of this review, an individual level selection outcome measure will require direct measurement of what was selected (e.g. not self-report). or example:

- from a vending machine, this will comprise a record of everything selected by the individual on that visit, or a record of items, such as chocolate bars, targeted in the intervention;
- in a restaurant or bar, this will comprise a record of everything selected by the individual for consumption on that visit, or a record of items, such as alcoholic or soft drinks, targeted in the intervention and selected for consumption on that visit;
- in a grocery store, this will comprise a record of everything selected by the individual on that visit, or a record of items, such as ready meals, targeted by the intervention and selected on that visit.

At a population level, selection with purchasing data will have to be derived from sales data supplied by the retailer from till receipts. Such data could be presented as selection of specific items or as total energy selected, calculated from the sales data presented. We will exclude studies that evaluate intention to select, without a measure of actual behaviour, or studies that measure hypothetical selection, or do not use real products, or both.

##### Consumption

We will consider healthier food and non-alcoholic drink consumption to be a reduction in energy consumed (total reduction in energy consumed, or lower consumption of higher energy products). Where total energy is available, we will prioritise this over lower consumption of higher energy products.

We will consider healthier alcoholic drink consumption to be a reduction in energy consumed (total reduction in energy consumed or lower consumption of higher energy products) or a reduction in the volume or units of alcohol consumed, or both.

Studies are required to assess consumption from real-world or laboratory settings by an objective measure, calculating the amount of a snack, meal or drink consumed by subtracting the amount of food or drink remaining after consumption from the amount served. This will be specified as either:

- amount of a food or drink consumed; or
- total energy consumed as part of a meal.

We will exclude studies that evaluate intention to consume without an objectively assessed measure of the behaviour.

Due to the nature of the intervention, we do not expect any significant health-related harms beyond those related to a change in behaviour in the unintended direction; that is, energy labels causing higher energy consumption, an outcome that would be captured by this protocol. We will, however, record all adverse events reported in the primary studies.

For all primary outcomes, we have no time point restrictions and will collect data on the longest outcome available while the intervention is still being applied. If there are multiple time points, we will privilege longer term data.

#### Search methods for identification of studies

We will run the search for studies on food and non-alcoholic drinks labelling from the search date (April 2017) of the original review on nutritional labelling. We will run the separate search for studies on energy labelling of alcoholic beverages – newly included for this update - from database inception.

#### Electronic searches

We will conduct electronic searches of these databases:

- Cochrane Central Register of Controlled Trials (CENTRAL) in the Cochrane Library;
- MEDLINE (OvidSP) (1946 to present);
- Embase (OvidSP) (1974 to present);
- PsycINFO (OvidSP) (1967 to present);
- Applied Social Sciences Index and Abstracts (ASSIA) from Cambridge Scientific Abstracts (CSA) (Proquest) (1987 to present);
- Science Citation Index (Web of Science Core Collection, Thomson Reuters) (1945 to present); and
- Social Science Citation Index (Web of Science Core Collection, Thomson Reuters) (1945 to present).

##### Grey literature

We will conduct electronic searches of these grey literature databases:

- Conference Proceedings Citation Index - Science (Web of Science) (1990 to present);
- Conference Proceedings Citation Index - Social Science & Humanities (Web of Science) (1990 to present).

We will also search trial registries for potentially relevant studies that are completed or in progress, using the World Health Organization (WHO) International Clinical Trials Registry Platform (ICTRP (apps.who.int/trialsearch/)), ClinicalTrials.gov (US National Institutes of Health Ongoing), and the EU Clinical Trials Register (www.clinicaltrialsregister.eu/), to identify registered trials.

Appendix 1 shows the MEDLINE search. We will impose no language restrictions. For the search for food products (including non-alcoholic drinks), we have modified slightly our search used for the 2018 version of this review (Crockett 2018). The changes largely involved removing lines of the search that related specifically to labels for fat, sugar or salt content, which are no longer an eligible intervention. We have developed a new search strategy for the separate search for alcoholic drinks.

We will upload all retrieved bibliographic records to EPPI-Reviewer for de-duplication before screening (Thomas 2020; see Selection of studies). Building on the corpus of studies included in our previously published Cochrane Review on nutritional labelling (Crockett 2018), we will also further develop, implement and evaluate a novel semi-automated study identification workflow powered by the Microsoft Academic Graph (MAG) dataset (Shemilt 2019; Sinha 2015), and hosted in MAG Browser (a new suite of tools in EPPI-Reviewer). The MAG dataset (updated every 10 days) currently comprises more than 240 million bibliographic records of research publications from across science, connected in a large network graph of conceptual and citation relationships. We are developing this MAG-enabled workflow in an ongoing methods research and development project that includes all published Cochrane Public Health Intervention Reviews. A preliminary analysis conducted for the latter project found that the MAG dataset has a recall of 0.90 for unique study reports included in published Cochrane Public Health Reviews (1591 of 1762), with the main gaps in its coverage identified as: (i) grey literature (not always indexed in MAG); (ii) conference abstracts (not always indexed in MAG); and (iii) trial registry records (not indexed in MAG).

In the current review, we will deploy the MAG-enabled workflow to automatically identify and prioritise ‘new’ (i.e. previously unseen) candidate, potentially eligible study reports (MAG records) and their corresponding full-texts for screening (see Selection of studies). We will also evaluate the MAG-enabled workflow by conducting a ‘Study Within A Review’ (SWAR) (All-Ireland Hub 2020). This embedded methodological study will compare: (a) use of MAG as a single source with ‘priority screening’ (see Selection of studies); and (b) use of conventional electronic search and screening methods via Boolean searches of multiple electronic databases (as described above and in Appendix 1). We will compare these methods in terms of their relative recall, precision, and screening workload. All data inputs to the SWAR will be automatically collected by EPPI-Reviewer.

We will also conduct forwards and backwards citation tracking of included studies, using MAG Browser in EPPI-Reviewer (in conjunction with the standard Microsoft Academic user interface (Microsoft 2020), with the aim of identifying further eligible study reports.

#### Searching other resources

We will search the websites of key organisations in the area of health and nutrition, including the following.

- Departments of Health for England (www.gov.uk/government/organisations/department-of-health), Scotland (www.gov.scot/Topics/Health), Wales (gov.wales/topics/health/?lang=en), and Northern Ireland (www.health-ni.gov.uk).
- European Commission (ec.europa.eu/commission/indexen).
- Centers for Disease Control and Prevention (www.cdc.gov).
- World Health Organization (who.int/en).
- National Institutes for Health Office of Disease Prevention (prevention.nih.gov).
- International Obesity Task Force (www.worldobesity.org).
- Institute of Alcohol Studies (http://www.ias.org.uk/Home.aspx).
- National institute of alcohol studies (https://www.niaaa.nih.gov/).
- Alcohol Change UK (https://alcoholchange.org.uk/).

### Data collection and analysis

#### Selection of studies

Two review authors will independently assess titles and abstracts of papers for inclusion, resolving any disagreements through discussion. Failure to reach consensus will lead to a discussion with a third review author. We will obtain full-text papers of potentially eligible studies identified during the first screening phase, and will assess them for inclusion using the same procedures as for titles and abstracts.

We will screen any further potentially eligible study reports identified by the MAG-enabled workflow (see Electronic searches) in EPPI-Reviewer using the same procedure described above, with the adjunctive use of ‘priority screening’ mode. In ‘priority screening’ mode, a machine learning algorithm, trained on all preceding screening decisions, is deployed to continuously reprioritise the order in which title-abstract records are assessed by screeners. In the MAG-enabled workflow, we will conduct the title-abstract and full-text screening stages in parallel when possible, exploiting links to online full-text sources in MAG records when available, using MAG Browser.

#### Data extraction and management

We will develop a data extraction form based on that used for the previous version of this review. Two review authors will independently pilot the draft to ensure that it enables reliable and accurate extraction of appropriate data. Two review authors will then independently extract all data on study characteristics along with results.

If a review author is also an author of an included study, a third reviewer will be involved in the data extraction process. Once the first phase of data extraction is complete, the first author will reconcile the two sets of data extraction forms. Where there are inconsistencies, the two data extractors will meet to discuss and reach a consensus. Where outcome data are missing or unclear, we will contact study authors. Finally, one author will enter the data into Review Manager 5 (Review Manager), and a second author will check the data entry. When multiple papers report data from the same study, we will treat the papers as one study. At outset, we intend to collect the data summarised below, which represent the maximum core dataset that we anticipate will be required based on our study eligibility criteria and logic model.

##### Study characteristics

- Study design
- Geographical setting: country
- Study (intervention) setting: restaurant (field); store (field); laboratory
- Product type: food; alcohol
- If applicable, selection with purchasing or selection without purchasing
- Duration of exposure
- Relationship between manipulated product and outcome (how outcome maps onto manipulated product)
- Relationship between manipulated product and other available products
- Concurrent intervention component in factorial design
- Concurrent intervention components confounded with comparison of interest
- Socioeconomic status context
- Summary ‘Risk of bias’ assessments
- Information on funding source and potential conflicts of interest from funding

##### Intervention characteristics

- Type of labelling used: calorie labelling; calories with PACE labelling; calorie labelling with other information about at least one other nutrient
- Label placement

##### Participant characteristics

- Age/age group
- Sex/gender (e.g. male, female)
- Ethnicity
- Socioeconomic status (e.g. occupational status; education; income; food insecurity; welfare receipt)
- Body mass index (BMI); body weight; body weight status
- Behavioural characteristics (e.g. dietary restraint; dietary disinhibition; level of intake or dependence, for targeted product)
- Biological state (e.g. hunger)

##### Outcome data

Should studies include more than one eligible measure of selection or consumption, we will use the measure of selection or consumption (pre)specified by the study authors as the primary outcome. If no primary outcome has been specified by study authors, we will use the measure of selection or consumption that accounts for the largest proportion of the overall diet. For example, if a study reported consumption measures of both total energy intake from a meal versus energy intake from a specific food only (e.g. a chocolate muffin), we would use total energy intake from a meal.

We will also use measures that relate to energy or alcohol content rather than mass or volume. If studies report comparable outcomes using a metric that is not a measure of energy (such as grams or millilitres), in consultation with the public health nutritionist on the review team (SAJ), we will determine if it is possible and appropriate to convert these to calories (for example, by using the formula presented in DeGroot 2012). For alcohol, if possible and appropriate, we will convert alcohol volume to alcohol content.

##### Assessment of risk of bias in included studies

Two review authors (NC, GJH) will independently assess the risk of bias for the primary outcomes for each included study. We will resolve any disagreements through discussion or by involving another review author (MP). We will assess risk of bias for RCTs using the RoB 2 tool (Sterne 2019) - using the accompanying Excel tool to manage the RoB assessments, and we will include a risk of bias table. This will assess the effect of the assignment to the intervention, for the following domains: bias arising from the randomisation process; bias due to deviations from intended interventions; bias due to missing outcome data; bias in measurement of the outcome; and bias in selection of the reported result. For cross-over trials, we will follow the supplement RoB 2 guidance for cross-over trials. This revised tool addresses bias arising from period and carryover effects (Higgins 2021a; Higgins 2021b). Should we encounter cluster-randomised controlled trials, we will follow the supplementary RoB 2 guidance for assessing risk of bias of these designs (Eldridge 2020). This revised tool includes assessing bias arising from the identification or recruitment of participants into clusters, and bias arising from the timing of identification and recruitment of individual participants in relation to timing of randomisation. For each study, we will judge each included outcome as ‘low risk of bias’, ‘some concerns’ or ‘high risk of bias’, according to these criteria:

- low risk of bias: when all domains are at low risk;
- some concerns: when one or more domains have some concerns, but none are at a high risk of bias;
- high risk of bias: when one or more domains are at high risk of bias, or multiple domains have some concerns to the extent that these reduce confidence in the results.

We will assess the risk of bias of controlled before-and-after (CBA) and interrupted time-series (ITS) studies using the ROBINS-I tool (Sterne 2016a), using the appropriate guidance (Sterne 2016b; Sterne 2020). This tool focuses on a specific result, uses a fixed set of domains of bias, and leads to an overall risk of bias judgement. For each study, we will specify a target trial (a hypothetical randomised trial whose results should be the same as the non-randomised trial under consideration). The effect of interest will be the effect of assignment to the intervention at baseline regardless of the extent to which the interventions were received (i.e. intention-to-treat). Before the tool is completed, we will specify whether the pre-defined critical confounders and co-interventions were identified in the study. Based on findings from the previous iteration of the review (Crockett 2018), and knowledge of the literature from the research team, at outset we consider the potential confounding domains to be key participant demographic characteristics (age, sex, and socioeconomic status distributions), and key study setting (intervention setting) characteristics (major events, different geographic areas, different retail environments). We consider possible co-interventions to be changes to labelling or other changes in packaging or any other interventions within the same physical environment (as classified by the TIPPME typology of these interventions (Hollands 2017)), or economic environment, such as price changes. We will assess the following domains: bias due to confounding; bias in selection of participants into the study; bias in classification of intervention; bias due to deviations from intended interventions; bias due to missing data; bias in measurement of outcomes; and bias in selection of reported result. This tool leads to an overall risk of bias judgement. Judgements for each bias domain, and for overall risk of bias, can be ‘Low’, ‘Moderate’, ‘Serious’ or ‘Critical’ risk of bias.

### Measures of treatment effect

Selection data can be either dichotomous (e.g. a choice with more versus less energy) or continuous (e.g. mean amount of energy purchased). We anticipate that consumption will typically be assessed using continuous data only (e.g. total or mean energy consumed) on the same measurement scale (i.e. kcal or data we can transform to kcal), and we will aim to calculate a mean difference (MD) with 95% confidence intervals (CIs) for each study when possible. If it is only possible to calculate a standardised mean difference (SMD) with 95% CIs because a different measure is used or the authors do not report the necessary data, we will calculate a standardised mean difference (SMD). If we encounter dichotomous data, we will report relative risk (RR) as the effect size (Mantel-Haenszel method). We will use a random-effects model to pool the data. In order to re-express effect sizes using a more familiar metric, we will calculate the percentage reduction in energy consumed over a typical meal, using an average of 600 kcal as a baseline. This amount is based on mean daily energy intake across the UK population of 1727 kcal or 7226 kJ (standard deviation (SD) 537 kcal or 2247 kJ, using data from the UK National Diet and Nutrition Survey (National Centre for Social Research 2012). Our approach to re-expressing effect sizes is based on that used in Hollands 2015 and Hollands 2019.

In all of the ITS studies, we will present the results as described by the study authors, typically as regression analyses. When studies also present data graphically, we will not attempt any re-analysis using segmented time series regression techniques if the data are already appropriately analysed by the study authors, or if we do not consider the study to be of sufficient quality to warrant re-analysis. If studies are not judged as having low risk of bias using the ROBINS-I tool (Sterne 2020), we will report them narratively (EPOC 2017).

### Unit of analysis issues

For cluster-randomised trials, where an analysis is reported that accounts for the clustered study design, we will estimate the effect on this basis, using reported test statistics (t-statistics, F-statistics or P values) to calculate standard errors if necessary. If this is not possible and the information is not available from the authors, then we will conduct an ‘approximately correct’ analysis by adjusting the actual sample size by the design effect in the meta-analyses. We will impute estimates of the intra-cluster correlation (ICC) using estimates derived from similar studies or by using general recommendations from empirical research. If the required information is not available, we will give the effect estimate as presented but report the unit of analysis error.

We will handle unit of analysis errors from studies with multiple intervention groups and cross-over trials in line with the relevant Cochrane guidance (Higgins 2021a). For studies with multiple intervention groups, we will combine groups that have used similar types of energy label and differ only in their presentational format, but will include interventions as separate comparisons if treatment groups comprise multiple eligible energy label types as per our pre-specified criteria. For studies contributing multiple comparisons,we will adjust the study weights to account approximately for the statistical dependencies between comparisons by dividing the sample size of the common intervention group as evenly as possible between the comparisons. As a post hoc sensitivity analysis, we will repeat the meta-analysis but instead will enter a single effect estimate for each multi-arm study using the mean SMD and mean variance across multiple comparisons from that study.

For cross-over trials that do not report data necessary for inclusion in the meta-analysis, a paired analysis will be approximated by imputing missing standard deviations where possible. We will follow the methods outlined in Chapter 23 of the *Cochrane Handbook for Systematic Reviews of Interventions* (Higgins 2021a).

### Dealing with missing data

We will include all data in the review using an intention-to-treat approach where possible. Where studies report dropouts or withdrawals, we will extract data on the number and any reasons for missing data using the Cochrane RoB 2 tool. We will attempt to obtain missing outcome data where possible by contacting authors.

#### Assessment of heterogeneity

In order to deal with inevitable methodological variability among studies that evaluate food or drinks consumed in real-world or laboratory settings, we will consider studies that evaluate labelling as an intervention and energy purchasing or consumption as outcomes similar enough to be meaningfully combined in meta-analyses of purchasing and consumption. We will assess clinical heterogeneity in participants, interventions and outcomes by comparing study characteristics across studies and applying pre-specified subgroup analyses to disentangle key characteristics (i.e. label type, study setting, SES) that could contribute to observed heterogeneity. We will assess statistical heterogeneity by visually examining the extent to which confidence intervals overlap and by formal statistical tests of homogeneity (Chi^2^) and measures of inconsistency (I^2^) and heterogeneity (Tau^2^). We will interpret the levels of heterogeneity made based on the recommendations of Deeks 2020.

#### Assessment of reporting biases

We plan to use funnel plots to identify small study effects, which in turn, could indicate publication bias. We will only use funnel plots if the included meta-analyses include more than 10 studies, based on the recommendations of Sterne 2019.

### Data synthesis

Where studies report a number of different types of interventions or outcome measures, we will follow the procedures described below.

- If treatment groups comprise multiple eligible energy label types as per our pre-specified criteria (e.g. energy label with PACE compared with energy label comprising calories), then we will include these two interventions as separate comparisons in the analysis. We will combine groups that have used similar types of energy label and differ only in their presentational format (e.g. PACE labels that display calories as walking and PACE labels that display calories as running will be combined).
- For included studies using factorial designs to investigate the effects of energy labelling as one of multiple intervention components, we will combine outcome data across groups to capture the main effect attributable to energy labelling. As previously stated, studies of only interventions with substantive concurrent components that are unrelated to but intrinsically confounded with energy labelling will be excluded.
- Where studies assess the impact of energy labelling adjacent to a range of food products and it is not possible to extract an effect summary for the range of food products, we will include data for the product representing the most complete meal; for example, sales of entrées/main meals (as opposed to sales of a side dish) (e.g. Dubbert 1984). If no products reasonably represent complete meals, we will extract data for products containing the greatest amount of energy.
- Where studies report a number of selection or consumption outcomes, we will use the primary outcomes specified by the authors. If an outcome is not specified as the primary outcome, we will prioritise the measure that accounts for the largest proportion of the overall diet. For example, if a study reported consumption measures of both total energy intake from a meal and the energy intake of a specific food (e.g. chocolate muffins), we would select total energy intake from a meal. If outcomes are reported that relate to increased consumption of lower energy foods and decreased consumption of higher energy foods, we will prioritise the latter.

We will analyse food (including non-alcoholic drinks) and alcohol studies separately, in line with other Cochrane Reviews on food and alcohol interventions (e.g. Hollands 2015; Hollands 2019). We will also analyse selection and consumption outcomes separately and will conduct separate meta-analyses for selection and consumption. We expect that we will mainly encounter studies where the public health goal is or aligns with a decrease in the energy consumed from the labelled food or drinks. Should we encounter any studies where the intervention aims to increase consumption of the labelled food in order to improve population health – such as foods beneficial to health including fruit and vegetables – then we will conduct a separate analysis for this outcome. A public health nutritionist on the review team (SAJ) will guide the assessment of this distinction.

We will use Review Manager 5 (RevMan 5) to perform meta-analyses, using the inverse variance approach for continuous data (Review Manager). For studies contributing multiple pairwise comparisons to a meta-analysis, provided the sample size was large enough, we will include each pairwise comparison separately. We will adjust the study weights to account approximately for the statistical dependencies between comparisons by dividing the sample size of the common group as evenly as possible between the comparisons. We will conduct meta-analyses only for the results of randomised controlled trials (RCTs), cluster RCTs and quasi-RCTs. We will combine cross-over trials that use an appropriate method of analysis (e.g. a paired analysis) with parallel design trials in the data synthesis and include in the meta-analysis. For cross-over trials that are not appropriately reported, we will approximate a paired analysis by imputing standard deviations where possible. We will not restrict analysis to the first period only. If dichotomous data are reported, we will discuss the most suitable approach for inclusion and will be guided by our statistician (MP). If we do identify and include cross-over trials and we find that pooled effect estimates from those studies suggest a systematic difference in effect size from parallel group studies, then we will exclude them in a sensitivity analysis (for example, if there is considerable heterogeneity - defined in the *Cochrane Handbook* as I^2^ of 75-100% [Deeks 2020] - due to a difference between cross-over and parallel groups). We will identify any concerns with specific cross-over trials (e.g. issues such as carryover effects) when assessing risk of bias for these types of studies using the modified RoB 2 tool (Higgins 2021a). As such, interpretation of analyses including these study designs will take account of risk of bias considerations, including within formal assessments of evidence certainty for any given outcome. Because of the increased risk of bias, we will summarise data from CBA and ITS studies in a narrative synthesis. In any cases where studies provide data that cannot be included in meta-analysis, then we will use an acceptable narrative synthesis method as described in the *Cochrane Handbook* (McKenzie 2020); for example, by using additional tables to present results in a systematic format. We anticipate that this will include at least the following variables: key participant, intervention and study characteristics, potential key modifiers (as specified by our planned subgroup analyses), comparison group, primary outcome, and results as reported by study authors. We will also provide summary effect estimates as reported in each of the papers not included in the meta-analysis (e.g. Table 3 in Crockett 2018).

### Subgroup analysis and investigation of heterogeneity

We will conduct the following subgroup analyses using a test of subgroup differences (Chi^2^) to determine the strength of evidence for possible effect modifiers and explore them as sources of heterogeneity.

- Environmental setting (e.g. restaurant, stores, laboratories).
- Type of energy label used (e.g. calorie labelling; calories with PACE labelling; calorie labelling with other information about at least one other nutrient)
- Socioeconomic status of participants (at the study level). Based on study authors’ explicit descriptions of the study sample and/or setting indicating that they sampled a population with specific socioeconomic characteristics indicative of relative deprivation (e.g. low or both high and low) either in terms of education, occupation, income, geographic location, or a combination. Where this information is not provided, we will assume that a high SES population was targeted. As in previous reviews (Hollands 2019), providing such data are available, our planned subgroups will be low; high; both low and high.

### Sensitivity analysis

We will repeat meta-analyses, including only studies judged to be at low risk of bias.

### Summary of findings table

We will prepare a summary of findings table, using GRADEpro software (GRADEpro GDT), that will describe the four main outcomes evaluated in the included RCTs or quasi-RCTs, as specified in the Primary outcomes:

- selection for food and non-alcoholic drinks;
- consumption for food and non-alcoholic drinks;
- selection for alcoholic drinks;
- consumption for alcoholic drinks.

If we encounter evidence from both RCTs and non-randomised studies for the same outcome, we will prioritise evidence from RCTs.

As specified in the *Cochrane Handbook* (Schünemann 2021), we will include the following sections in the summary of findings table.

- The population and setting addressed by the available evidence.
- The experimental and comparison interventions (energy label (intervention) versus no energy label (control)).
- The health outcomes.
- The illustrative risk of each outcome (risk with no labelling and risk with calorie labelling).
- The absolute and relative effects for each outcome.
- The number of participants and studies contributing to the analysis of each outcome.
- GRADE assessment for the certainty of the evidence for each outcome.
- Comments.
- Explanations.

Using the GRADE (Grading of Recommendations, Assessment, Development and Evaluations) framework (Guyatt 2011), we will assess the certainty of each body of evidence relating to primary outcomes that are incorporated into summary estimates of effect. Two review authors (NC, GJH) will independently assess the certainty of each body of evidence using the GRADE framework. We will resolve any disagreements through discussion or by involving another review author (MP). We will consider risk of bias, precision of results, directness, consistency and publication bias in the GRADE assessments. For the risk of bias component, we will assess risk of bias across studies using the RoB 2 tool for randomised studies and the ROBINS-I tool for non-randomised studies (Schünemann 2019). Each body of evidence will be given a GRADE rating. There are four standard GRADE levels of certainty - high, moderate, low and very low. We will assign to the evidence from RCTs and non-randomised studies an initial certainty rating of high and levels will be downgraded accordingly. We will report the degree of certainty assigned for each outcome, along with justification for the decisions, in the Results. Justifications underpinning GRADE assessments will also be included in the summary of findings tables.

## Supplementary Material

Supplementary Appendix

## Objectives

- To estimate the effect of energy labelling for food (including non-alcoholic drinks) and alcoholic drinks on healthier selection (with or without purchasing) and consumption.
- To assess possible modifiers – energy label type, setting and socioeconomic status - of the effect of energy labelling on selection and consumption of food and alcohol.
