# Supplementary material for: Energy (calorie) labelling for healthier selection and consumption of food or alcohol
Source: Cochrane Database Syst Rev. Author manuscript; Available in PMC 2021 Jun 30. (PMC8194387; doi:10.1002/14651858.CD014845)
Supplement: Supplementary Appendix [file EMS127935-supplement-Supplementary_Appendix.docx]

**Appendix 1**

**MEDLINE search 1: for food products (including non-alcoholic drinks)**

1 exp Food packaging/ and (label$ or content$ sign$ or symbol$ or ticket$ or sticker$ or diet$ or health$ or calori$ or nutritio$ or guideline daily amount$ or recommended daily amount$ or nutrient reference value$ or nutrient daily value$).ti,ab.

2 food pack$.ti,ab.

3 exp Product labelling/ and (food$ or diet$ or health$ or calori$ or nutritio$ or guideline daily amount$ or recommended daily amount$ or nutrient reference value$ or nutrient daily value$ or snack$ or eat$).ti,ab.

4 exp Food Labeling/

5 ((nutritio$ or nutrient$) adj5 (label$ or content$ sign$ or symbol$ or ticket$ or sticker$)).ti,ab.

6 (nutrition$ information or nutrient$ information).ti,ab.

7 (Food$ label$ or food$ content$ label$ or food$ content$ sign$ or food$ content symbol$ or food$ content$ tag$ or food$ content$ ticket$ or food$ content$ sticker$).ti,ab.

8 traffic light$.ti,ab

9 (guideline daily amount$ or nutrient reference value$ or nutrient daily value$).ti,ab.

10 (recommended dietary allowance$ adj5 (label$ or content$ sign$ or symbol$ or information or ticket$ or sticker$)).ti,ab.

11 ((Calorific or calorie$ or caloric) and (label$ or content$ sign$ or symbol$ or ticket$ or sticker$)).ti,ab.

12 ((Calorific or calorie$ or caloric) adj information).ti,ab.

13 (menu and (label$ or content$ sign$ or symbol$ or tag$ or ticket$ or sticker$)).ti,ab.

14 (menu and (nutritional content$ or nutritional information or traffic light or guideline daily amount or GDA or healthy choice or calorie)).ti,ab.

15 (Label$ adj2 (legislation$ or regulation$ or policies or policy)).ti,ab.

16 Healthy choice.ti,ab.

17 exp Product labelling/ and (drink? or beverage? or soda? or flavo? red water? or fruit water? or cordial? Or squash? or juice? or smoothie? or milkshake? or tea or teas or coffee?).ti,ab.

18 (Drink$ label$ or Drink$ content$ label$ or Drink$ content$ sign$ or Drink$ content symbol$ or Drink$ content$ tag$ or Drink$ content$ ticket$ or Drink$ content$ sticker$).ti,ab.

19 ((drink? or beverage? or soda? or flavo?red water? or fruit water? or cordial? or squash? or juice? or smoothie? or milkshake? or tea or teas or coffee?) and (label$ or content$ sign$ or symbol$ or ticket$ or sticker$)).ti,ab.

20 1 or 2 or 3 or 4 or 5 or 6 or 7 or 8 or 9 or 10 or 11 or 12 or 13 or 14 or 15 or 16 or 17 or 18 or 19

21 exp Food Preferences/

22 exp Food Habits

23 exp Feeding Behavior/

24 exp Eating/

25 exp Diet/

26 exp Choice Behavior/

27 (intak$ or consume or consumes or consumption or consumed or eat$ or diet$).ti,ab.

28 (food adj5 (preference$ or habit$ or behavio?r$ or choice$ or decision$ or decid$ or inclin$ or lik$ or choos$ or select$ or pick$)).ti,ab.

29 ((drink? or beverage?) adj5 (preference$ or habit$ or behavio?r$ or choice$ or decision$ or decid$ or inclin$ or lik$ or choos$ or select$ or pick$)).ti,ab.

30 21 or 22 or 23 or 24 or 25 or 26 or 27 or 28 or 29

31 exp Restaurants/

32 (purchas$ or buy$ or sale$ or vend$ or sell$).ti,ab.

33 (shop$ or store$ or supermarket$ or market$ or outlet$ or retailer$ or point of purchase).ti,ab.

34 (restaurant$ or cafe$ or bar$ or canteen$ or cafeteria$ or dinner hall$ or dining area$ or dining room$ or refector$ or eatery or mess or buffet or bistro$ or eating place$).ti,ab.

35 31 or 32 or 33 or 34

36 20 and (30 or 35)

37 limit 36 to dt=20170425-YYYYDDMM

**MEDLINE search 2: for alcoholic drinks**

1. exp Alcoholic Beverages/

2. (dr#nk* or beverage* or alcohol* or beer* or lager* or wine* or cider*).ti,ab.

3. 1 or 2

4. exp Product Labeling/

5. ((alcohol* or drink*) adj5 (unit? or guideline* or standard drink*)).ti,ab.

6. ((calorie* or nutrition* or energy or ingredient*) adj5 (label* or inform* or menu* or poster* or glass* or beermat* or bottle* or packag*)).ti,ab.

7. 4 or 5 or 6

8. exp Alcohol Drinking/

9. ((purchas* or pour* or select* or consum*) adj5 (alcohol* or drink* or beer* or wine* or lager* or cider*)).ti,ab.

10. exp Health Knowledge, Attitudes, Practice/

11. exp Energy Intake/

12. exp Consumer Behavior/

13. 8 or 9 or 10 or 11 or 12

14. 3 and 7 and 13
